# Scalability of API-Loaded Multifilament Yarn Production by Hot-Melt Extrusion and Evaluation of Fiber-Based Dosage Forms

**DOI:** 10.3390/pharmaceutics16081103

**Published:** 2024-08-22

**Authors:** Christoph Rosenbaum, Naemi Gerds, Liliane Hack, Werner Weitschies

**Affiliations:** Department of Biopharmaceutics and Pharmaceutical Technology, Institute of Pharmacy, University of Greifswald, Felix-Hausdorff-Straße 3, 17489 Greifswald, Germany

**Keywords:** spinning techniques, microfibers, knitting techniques, drug release, hot-melt extrusion, continuous manufacturing

## Abstract

Fiber-based technologies are widely used in various industries, but their use in pharmaceuticals remains limited. While melt extrusion is a standard method for producing medical fibers such as sutures, it is rarely used for pharmaceutical fiber-based dosage forms. The EsoCap system is a notable exception, using a melt-extruded water-soluble filament as the drug release trigger mechanism. The challenge of producing drug-loaded fibers, particularly due to the use of spinning oils, and the processing of the fibers are addressed in this work using other approaches. The aim of this study was to develop processes for the production and processing of pharmaceutical fibers for targeted drug delivery. Fibers loaded with polyvinyl alcohol and fluorescein sodium as a model drug were successfully prepared by a continuous melt extrusion process and directly spun. These fibers exhibited uniform surface smoothness and consistent tensile strength. In addition, the fibers were further processed into tubular dosage forms using a modified knitting machine and demonstrated rapid drug release in a flow cell.

## 1. Introduction

Yarns and fibers have been an important technology for humans for many centuries, and breakthroughs such as the spinning jenny (1765), combined with the steam engine, were a major cause of the start of the Industrial Revolution [1]. The potential uses for fibers and yarns seem almost limitless, although yarn, which has become a truly high-tech product in recent decades, is generally only an intermediate product [2]. From knitting, embroidery, crochet and weaving to felting, a wide range of processing technologies have been developed in recent decades to take yarns further.

Many fiber-based concepts with various polymers have also already been described in the biomedical field, illustrating the great potential of these approaches [3,4,5,6]. In the field of pharmaceutical development, electrospinning has been established for several years as a means of producing fiber components containing active ingredients that can be inserted into wounds, placed on the eyes or even swallowed as novel dosage forms [7]. Electrospinning technology generally enables the production of very fine fibrous nonwovens that can then be cut or punched into suitable surface shapes [7,8]. Similar to solvent casting, electrospinning involves the preparation of a polymer solution containing an active ingredient. This is then pressed through a thin needle into a high-voltage field, where the strong field ensures that the polymer solution is accelerated accordingly and deposited in a fleece-like manner in the finest structures on the other side of the pole. Systems of various sizes to produce fiber components for medical purposes already exist, and very large systems with corresponding production volumes have been described [9,10,11,12].

In pharmaceutical applications, solvent-based electrospinning is used to produce fibers containing active ingredients, whereas in other fields, such as the plastics and textile industries, the solvent-free process of melt extrusion is widely used [13]. In melt extrusion, spinning oils are used to prevent static charging of the individual fibers in a multi-stage batch process [13,14]. Such spinning oils are washed out with suitable solvents at the end of the batch process. However, the spinning oils themselves represent a regulatory challenge for fibers used for pharmaceutical purposes, and the step of washing the fibers to remove the spinning oils, which can wash out potential active ingredients from the fibers, must also be critically evaluated [15]. There are only a few publications, for example, on monofilament sutures containing triclosan to promote wound healing. However, as a medical device, these sutures work primarily through their mechanical properties rather than pharmacological effects [16,17,18,19,20].

The almost unlimited possibilities for further processing of the spun fibers using innovative, structuring production processes and, of course, the different regulatory requirements compared to the pharmaceutical industry are the driving forces behind the plastics and textile industries’ preference for melt extrusion [2,14]. The EsoCap system is one of the few described dosage form concepts containing a rapidly water-soluble but drug-free filament produced by melt extrusion, which serves as a trigger mechanism for the placement of the actual film-based dosage form [15,21]. The aim of this work was to investigate the feasibility of producing drug-loaded fibers by continuous melt extrusion without the use of spinning oils and to investigate subsequent processing steps to produce model dosage forms. In addition, the study will identify the challenges and potential of fiber-based dosage forms for further biopharmaceutical application concepts.

## 2. Materials and Methods

### 2.1. Materials

Parteck MXP 4-88 polyvinyl alcohol (32,000 g/mol) was purchased from Merck (Darmstadt, Germany). Fluorescein sodium was chosen as an easy to analyse model substance and was purchased from Sigma-Aldrich Chemie (Darmstadt, Germany). However, it does not currently have any significant biopharmaceutical relevance for future fiber-based applications. Potassium dihydrogen orthophosphate was obtained from neoFroxx GmbH (Einhausen, Germany), and sodium hydroxide was purchased from AppliChem GmbH (Darmstadt, Germany).

### 2.2. Methods

#### 2.2.1. Hot-Melt Extrusion and Spinning Process

**Figure 1:**
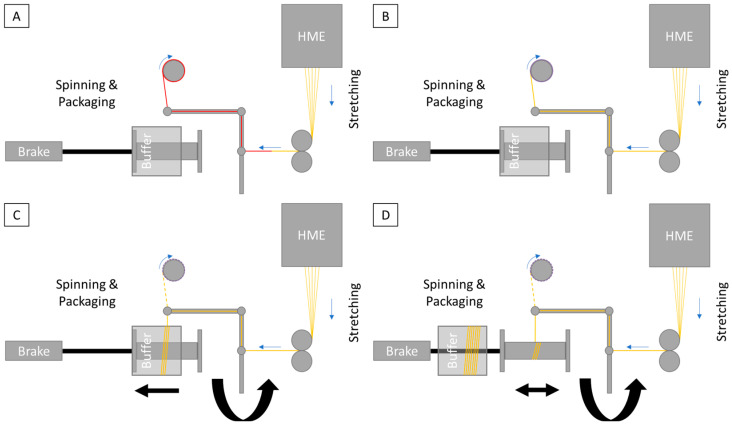
Schematic representation of the structure of the system to produce active-ingredient-loaded fibers and the steps for starting up the system. (**A**): Threading of the drawn fibers through the spinning device by means of a previously inserted polyester thread. (**B**): Continuous winding of the active-ingredient-laden fibers onto a reel arranged outside the spinning process and further acceleration of the drawing of the fibers up to the target thickness. (**C**): Starting up and accelerating the actual spinning machine to the target speed and depositing the fibers that have not yet been drawn onto a buffer tube. The fibers are cut up to the reel so that the fibers deposited there are not unwound. (**D**): As soon as the yarn is completely twisted, the buffer tube can be pulled over the spool so that the yarn can be deposited in parallel onto the finished product spool. The yarn between the buffer tube and the spool is also cut off.

The unit to produce fibers and yarns can basically be split into three different areas: hot-melt extrusion (HME), stretching and spinning (Figure 1). The hot-melt extrusion line consists of the Three-Tec ZD 9 FB feeder (Seon, Switzerland), to ensure uniform material feeding to the ZE12 twin-screw extruder with segmented screw. Due to moisture problems, the feeder and transition to the extruder were encapsulated, and desiccant was added inside the capsule. Moxx thermohygrometers (TFA Dostmann, Wertheim, Germany) were installed in the encapsulation to monitor the humidity inside the capsule, which was less than 20% RH. The ZE12 twin-screw extruder used (ThreeTec, Seon, Switzerland) was equipped with water inlet cooling. A stainless steel multifilament spinneret with a 90° deflector was added; the device was manufactured (Protiq, Blomberg, Germany) using 3D powder bed fusion (PBF). The feeding rate during the standard manufacturing process was 1%, and the number of revolutions per minute of the segmented screws was 150, while the following temperatures were applied in the individual segments: inlet 20 °C; zone 1: 80 °C; zone 2: 150 °C; zones 3 and 4 and the deflector with the nozzle: 165 °C. A table with all parameters of the process can be found in the Supplementary Materials (Table S1).

A 100 × 473 complete V2 conveyor belt from Three-Tec (Seon, Switzerland), modified with a stainless-steel pressure roller, was used to stretch the extrudate. All components required for assembly were designed in house and manufactured from PLA using 3D printing. The take-off speed of the take-off belt used was 7.5 m/min during fiber production. However, higher take-off speeds of 15.0 m/min have also been tested; the parameters are given in Table S1.

The SpinningRosi NG (Next Generation) apparatus was built in house and represents a systematic further development of the previous spinning apparatus [15]. Similar to the structure of the SpinningRosi, there is first a module for spinning the stretched fibers, which is controlled by a Nema 23 stepper motor (Stepperonline, OMC Corporation Limited, Nanjing, China). When the system is initially activated, the extruded and partially stretched fiber material (not yet at the final speed) is knotted with a polyester yarn (Figure 1A in red), which is attached to a take-up roll driven by a NEMA 17 stepper motor (Stepperonline, OMC Corporation Limited, China) via a belt drive. The purpose of the take-up roller is to pull the polyester yarn with the knotted extrudate continuously through the spinning machine before the spinning process starts. To ensure easy start-up of the system, constant fiber guidance or fiber tension and the ability to operate the system without spinning oils, the belt driving the take-up roll is designed for maximum elongation slip. This ensures that the newly extruded and spun material is constantly removed, but the tension does not damage the fine fiber components. The start-up speed of the take-up roll without slip was 450 rpm, giving a maximum tested speed of 56.5 m/min with a roll diameter of 40 mm.

The task of the maximum slip belt coupling was to bring the fiber stretching to the desired target speed without further adjustments to the system and without causing any uncontrolled behavior in the handling of the fibers. As soon as the fiber material had reached the target speed because of the stretching process, the spinning module could be started, and the fiber material deposited on a special sleeve called a buffer tube.

The buffer, a ball-bearing sleeve that rotated with a slight delay as the spool of the spinning unit did, served to prevent unspun fiber material from being deposited on the final spool, which was intended to hold only the finished product. This allowed the material to accumulate on the buffer until the extruded fibers were twisted by the increasing speed of the spinning unit. The spinning system, driven by a non-slip toothed belt, gradually increased its speed, reaching 560 rpm during the standard yarn production process. Once the extruded and spun fiber material was fully twisted, a standard spool (E-80) could be inserted through the buffer tube. The buffer tube was then removed, allowing the spun fiber to be wound onto the spool, which was then fully loaded with the finished material. To ensure uniform parallel winding of the yarn, the spool was moved back and forth consistently under the spinning module. Both the buffer tube and the final product spool were slowed down using a simple thread brake with a force of 0.5 N to allow for continuous material transport within the spinning unit.

**Figure 2:**
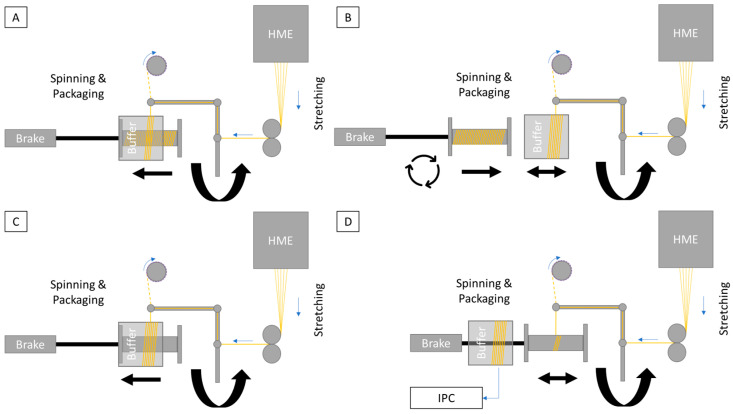
Schematic illustration of the production process, particularly the spool changes during continuous system operation. (**A**): When the product spool is filled with material, the buffer tube is moved from the side and placed over the product spool, so that the yarn can now be wound onto the buffer tube. In the next step, the product spool is removed from the spinning system through the buffer tube. (**B**): The product spool is replaced while the spinning system continues to deposit material onto the buffer tube. As soon as the empty spool is in place, it is reintroduced into the process. (**C**): Once the new spool is set up, the buffer tube can be pulled out of the spinning system in the same manner as during system startup, allowing the yarn to be wound onto the product spool again. (**D**): The material on the buffer tube can be used for further processing, including destructive in-process tests such as tensile strength measurement.

Once the spool in use was fully loaded, the buffer tube was moved back over the spool, shifting the thread from the spool onto the buffer tube (Figure 2A). This allowed the full spool to be removed from the system through the tube, enabling a spool change and the reintroduction of an empty spool into the process without interrupting the continuous manufacturing and spinning process (Figure 2B). After the empty spool was repositioned in the system, the buffer tube could be pulled out of the spinning module, allowing the yarn to be placed back on the spool (Figure 2C). The material on the buffer tube is also ideal for quality assurance measures, such as in-process checks (Figure 2D).

#### 2.2.2. Tensile Strength, Elongation, Knot-Pull Tensile Strength

The yarns were tested for tensile strength, elongation and knot strength using the Texture Analyzer TA plus and the associated software Nextgen Plus version 3.0 (Ametek, Berwyn, PA, USA). The yarn material was placed between two clamping blocks 50 mm apart, and the resulting force was measured at a constant pull-out speed of 100 mm/min. To measure the knot-pull tensile strength, an overhand knot was tied before the yarn was placed between the clamping blocks of the texture analyzer.

#### 2.2.3. Knitting Processing of Fibers Containing Active Ingredients

A semi-manual six-needle AddiEgg knitting machine (Gustav Selter GmbH & Co. KG, Altena, Germany), originally designed for toy applications, was used to produce advanced fiber-based dosage forms from the extruded, spun and drug-loaded yarns. The knitting machine was driven by a NEMA 17 stepper motor (Stepperonline, OMC Corporation Limited, China). A weight provided by the machine manufacturer maintained a constant yarn tension in the knitted fabric. The fabric spool from which the yarn was unwound was also driven by a NEMA 17 stepper motor (Stepperonline, OMC Corporation Limited, China) to prevent tension variations due to spool inertia. A duck yarn brake (Hans Kraus Webereibedarf.de, Albstadt, Germany) with adjustable stainless steel plates ensured stable yarn tension prior to the manufacturing process.

#### 2.2.4. Optical Analysis

A Stemi 2000-C microscope (Carl Zeiss, Jena, Germany), a SteREO CL 1500 ECO lighting system (Carl Zeiss, Germany) and an AxioCam Icc 1 camera system (Carl Zeiss, Germany) were used for the optical assessment of the fiber-based dosage forms produced. Image processing was carried out with AxioVS40 V 4.8.2.0 software (Carl Zeiss, Germany).

#### 2.2.5. Modified USP 4 Dissolution Testing

A standard USP 4 (flow-through cell) was used for dissolution testing. This was modified so that, prior to dissolution testing, the tubular test objects were placed on an open-pored cylindrical sponge 40 mm long and 10 mm thick. This was attached to the sieve insert of the flow cell with a needle and placed in the center of the flow cell. This arrangement was intended to prevent the highly water-soluble tubular structure of the object from collapsing on first contact with water during the dissolution tests and forming a depot-like, highly viscous polymer solution at the bottom of the flow cell. Dissolution tests were performed at a flow rate of 16 mL/min in 500 mL phosphate buffer 7.4 USP at 37 °C. The amount of drug released was measured online in the Schott bottles used as reservoirs. The samples were analyzed spectrophotometrically using a fiber-optic-based system (Cary^®^ 60, Agilent Technologies, Santa Clara, CA, USA) equipped with 10 mm slits at 491 nm against a baseline correction at 600 nm. The absorbance maxima and linearity of the absorbance range were determined. The analytical method was validated for linearity, precision, accuracy and selectivity.

#### 2.2.6. Film Tube Preparation

The dissolution tests were carried out on the melt-extruded tubular mesh-like samples and on two film tubes of different thicknesses produced by solvent casting technology [22]. The polymer films were prepared using 29.85 g Parteck MXP 4-88 polyvinyl alcohol, with 70.0 g purified water as the solvent. As a model drug, 0.15 g fluorescein sodium (FS) was added. All components were mixed with a magnetic stirrer at 500 rpm in a laboratory glass bottle and then heated in a water bath at 80 °C for one hours with continuous stirring at 100 rpm. For reasons of stability of the API, the following steps were carried out in the absence of light. The mixture was stirred at 50 rpm overnight and cooled down to produce a bubble-free polymer mixture. If the mixture was not completely free of air bubbles, it was centrifuged (Centrifuge 5702 R, Eppendorf SE, Hamburg, Germany) at 4400 rpm for 15 min at 20 °C to obtain a homogeneous and bubble-free mixture.

The prepared polymer solution was then applied to a coating knife (mtv messtechnik oHG, Erftstadt, Germany) and coated using a coating bench (Automatic Precision Film Applicator CX4, mtv messtechnik oHG, Erftstadt, Germany). It was applied homogeneously to the polyethylene-impregnated side of a siliconized coated liner using a square applicator (gap height: 500 µm, 850 µm). The film was dried overnight at room temperature, and then the large film strips were cut into elongated pieces of 40 mm and glued with an overlap of about 4 mm to a round film with an inner diameter of about 10 mm using a very small amount of water. After gluing, the film tubes were cut into lengths of 40 mm and stored in reaction tubes at room temperature in the absence of light.

## 3. Results and Discussion

### 3.1. API Ingredient Fiber Production and Spinning

**Figure 3:**
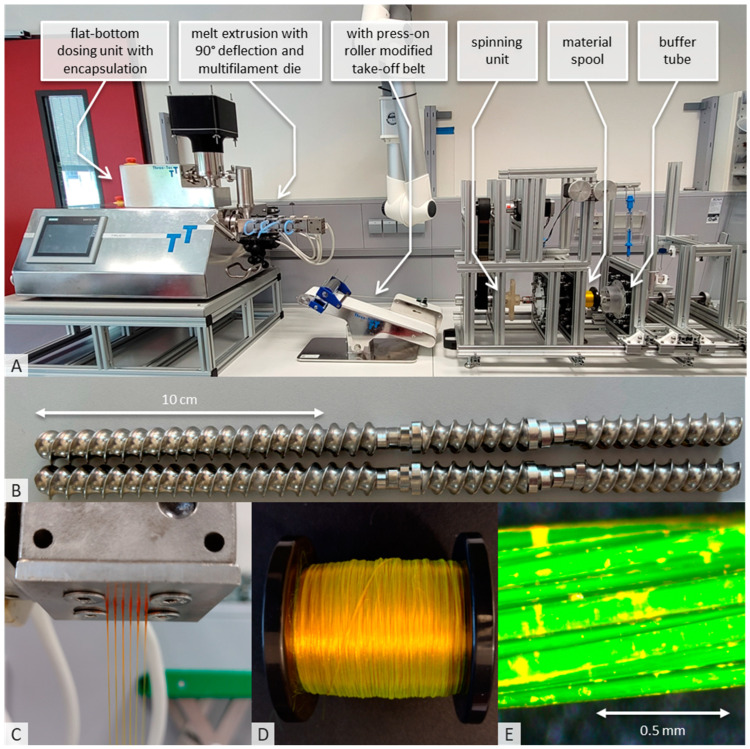
(**A**): Structure of the process to produce polymer yarns containing active ingredients by hot-melt extrusion, consisting of the melt extrusion line, a modified take-off belt and the spinning machine itself. (**B**): Geometry of the segmented screws used in the extrusion line. (**C**): Discharge of the API melt from the multifilament die. (**D**): Spun and wound polymer fiber on the standard spool (E80) used. (**E**): Microscopic image of the melt-extruded polyvinyl alcohol yarn with fluorescein sodium.

The flow properties of the dried powder mixture containing the active ingredient were ideal for feeding into the melt extrusion system using the flat-bottom feeder. The encapsulation of the system and the desiccant used ensured that the humidity above the feeder hopper remained consistently below 20% RH (Figure 3A). After a ramp-up time of approximately 30 min, due to the low load on the hot-melt extrusion (HME) system, a bubble-free and clear extrudate (Figure 3C) with an optimum viscosity for manual stretching was produced. This extrudate was then fed to the modified take-off belt. At a low stretching speed of 1.5 m/min, the material was stretched until it was sufficiently uniform and thin to be knotted with the polyester yarn fed through the spinning unit. The electric motor, connected to the take-up roller via a slipping coupling, moved the material quickly through the system, preventing uncontrolled material feed and ensuring that the material was not damaged. The system was also easily increased to the planned target speed of 7.5 m/min. In subsequent tests, the process was easily doubled to 15 m/min by adjusting the melt extrusion system’s conveying capacity, which was the maximum speed of the take-up belt.

It was also possible to start spinning on the buffer tube and then switch to the final bobbin. However, manual separation of the fibers between the two spool components required some skill. During spinning, the fibers were twisted at 560 rpm to produce a uniform, homogeneous, yellowish-green thread, which was wound evenly in parallel layers onto the bobbin over several hours (Figure 3D). Microscopic examination of the thread showed no structural abnormalities or inconsistencies, and the thread had a very smooth and uniform surface (Figure 3E). Fluorescein sodium was chosen as the active pharmaceutical ingredient (API) because of its high melting point (over 300 °C when decomposed) and its suitability for optical study and analytical accessibility, although it has no therapeutic benefit in this context. A diameter of 80 µm was measured using a microscope for one of the 32 fibers in the yarn. The prototype system demonstrated increased performance with error-free processing up to a speed of 9 m/min at a constant running speed of 800 rpm. Above this speed, there was no additional performance reserve due to system vibration.

The modified hot-melt extrusion system we have developed, with a multi-filament die, simple stretching and spinning and an oil-free direct spinning process, enables solvent-free, single-stage processing of drug-containing powder blends into drug-loaded spun fibers on a laboratory scale. The system is also capable of continuous solvent-free production of larger quantities of fibers, up to several kilograms per day, directly from powder. Further improvements, in particular the optimization of components to reduce the internal friction of the yarn guide, could improve performance on a production scale.

As previously described, the switch from buffer tube to finished spool involved considerable uncertainty and required a certain level of operator skill. In the future, integrated hot cutting systems could provide an efficient alternative, simplifying the changeover process and reducing the reliance on operator skill. In addition, the challenge of switching between the buffer tube and the product spool was due to the inertial mass of each component. This mass inertia meant that an abrupt acceleration of the unit, driven by the polymer thread, was required when changing between media. An alternative to using a yarn brake with a force transducer to decelerate the spool or buffer tube could be a direct drive system with a defined target speed and corresponding control loops. This approach would accelerate both the buffer tube and the test spool, accordingly, significantly reducing the force on the yarn during start-up and preventing any weakening of the material. Future development should include monitoring the yarn tension between stretching and entry into the spinning unit as an in-process control. This would allow the acceleration or deceleration of the product bobbin or buffer tube to be easily controlled based on yarn tension to ensure consistent yarn quality. The yarn on the buffer tube could also be used for destructive in-process checks, such as tensile strength or elongation tests. In addition, a visual inspection system, such as a camera or shadow-based device, could be used to monitor the quality of the extrudate from the die and provide early detection of fiber production defects. Alternatively, online measurement of fiber thickness during processing, based on the excipients and APIs used, would be valuable. Fiber thickness can substantially influence the release behavior and overall performance of API-containing dosage forms.

### 3.2. Tensile Strength as a Measure of Extruded Material Quality and Homogeneity

**Figure 4:**
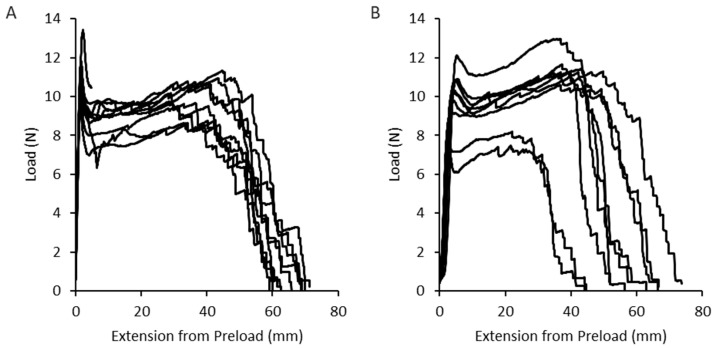
Individual values of the tear strength and elongation test results in a force–displacement diagram at a tensile rate of 100 mm/min. (**A**): Standard tensile test. (**B**): Knot tensile strength.

To investigate the homogeneity of the fiber quality, the tensile strength and elongation of the yarns were measured using the Texture Analyzer. The standard tensile strength of the samples measured averaged 11.2 ± 1.0 N and showed a relative elongation of 131 ± 9% (Figure 4A). In addition to the actual tensile strength, the knot tensile strength was also determined. The purpose of this test was to assess the suitability of the fibers containing active ingredients for further processing in the knitting process. In this case, the tensile strength at tight radii caused by a knot was used. The knot used was the basic knot, also known as the overhand knot. These tests showed a slight decrease in the tensile strength of the material and an increase in the scatter of the results, resulting in a load of 11.0 ± 2 N and an elongation of 112 ± 20% (Figure 4B). These results are broadly consistent with the expectation that the tight radius caused by the knot would lead to increased stress on the polymer material, resulting in premature material fatigue and a higher standard deviation. Nevertheless, the tensile strength of over 10 N is a very favorable basis for further processing of the fibers by means of a knitting process. It should be noted that the fiber materials tested were not tested for tensile strength and elongation immediately after manufacture. During the manufacturing process, the fiber samples were stretched accordingly, placed in polyethylene bags with desiccant and stored at room temperature in the absence of light. Up to one week could elapse between production and measurement of tensile strength and elongation. As shown in previous studies, the moisture absorbed by the polymer is the cause of the exceptional extensibility of the polyvinyl alcohol filaments [15]. In addition, as already shown by Ghosal et al., fiber-based objects exhibit significantly greater flexibility than comparable films produced by solvent casting [23]. 

The thin fibers obtained by melt extrusion allow exceptional flexibility compared to polymer films of comparable weight. Without the addition of a plasticizer, the films containing active ingredients were able to exhibit a flexibility that could possibly have been achieved with a polyvinyl alcohol film with a plasticizer content of 5–10% glycerol relative to the solid content, where the plasticizer, in turn, could have influenced stability and release.

### 3.3. Knitting of Fibers Containing Active Ingredients

The major advantage of spun yarns containing active ingredients, compared to fibers produced by methods such as electrospinning, is the variety of shapes, structures and functionalities that can be achieved with intermediate product yarns—features that are already being exploited in the textile industry today [24]. The melt extrusion process therefore offers a wide range of possibilities for adaptation to specific requirements, beyond the semi-automatic knitting process described here for the production of a tube. The knitting machines tested—a French Knitting Machine (4-pin, metal), a Sentro Knitting Machine (22-pin, plastic) and an AddiEgg (6-pin, plastic)—differed in size, material properties and needle shape. For example, the French knitting machine used initially, with its metal needles, presented major challenges in terms of mechanical stability, affecting the continuous production of the knitted fabric.

**Figure 5:**
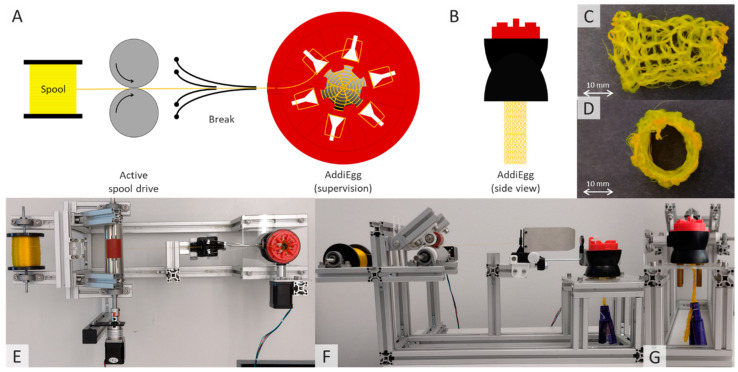
(**A**): Schematic arrangement of the knitting process from left to right: The spool loaded with the extruded drug loaded yarn is clamped and unwound via driven rollers so that no forces can be transferred from the unwinding of the yarn to the knitting process. This is followed by a brake with which the yarn tension can be adjusted at the inlet of the AddiEgg knitting unit, which is also motorized. (**B**): The knitted fiber tube is pulled downwards out of the knitting unit by a weight. (**C**,**D**): Exemplary sections of the knitted fibers that have been separated and simultaneously fused with a hot air dryer. (**E**): Top view of the manufacturing process. (**F**,**G**): Side view of the manufacturing process.

The internal thread of the metal knitting needles rubbing against the fiber material caused the material to tear even at low mechanical tensile loads, making continuous production impossible. In this context, further tensile tests could be carried out to determine the abrasion within the knitting tool, but also in advance within the twisting system to determine the suitability for other yarns [25]. On the other hand, the use of the large Sentro knitting machine, with its 22-needle design, made it possible to produce very large textile-like surfaces that could be used for dermal applications. To produce small model dosage forms, all tests were carried out using the Addy Egg knitting machine (6-pin, plastic needles). To standardize the process, the knitting machine was driven by an electric motor (Figure 5). The yarn spool was also driven to prevent the knitting process from unnecessarily stressing the yarn material. Further stretching of the material during the process was to be avoided, which could have led to an inhomogeneous drug load per area of the knitted objects. 

Once an endless tube had been produced, it was threaded onto a metal tube. The tube was then cut into equally sized segments using a hot air dryer at 200 °C and minimal airflow with a 3 mm nozzle. The hot air process not only served as a simple separation process, but also allowed the end fibers to be welded together at the same time, producing structurally stable and rib-free fiber components while retaining the fine fiber structure (Figure 5C,D).

However, the knitting process described is only the beginning of a further development of fiber or textile-like dosage forms for drug release. The general use of fibers instead of, for example, film-based dosage forms can already offer advantages in terms of solubility and dissolution rate, for example, due to the large surface area, depending on the requirements [26,27]. The tubular dosage form produced here is only a simply structured, textile-based dosage form, although processes to produce smart textiles are also described in the literature [24,28,29,30,31]. However, the full potential of the dosage form has not yet been exploited, a combination of fibers containing active ingredients and innovative manufacturing technologies could therefore contribute to modern pharmacotherapy in the future. The adaptation of textile structures using special fabric designs, as used in smart textiles, and the use of shape memory polymers offer great potential for further development and innovation [26,27,28,32,33,34,35,36].

Corresponding mechanically modifiable structures, which could, for example, help to homogenize the small intestine passage times of dosage forms according to the physiology of the body, could offer considerable added value, particularly for active ingredients with a short absorption window [37]. In principle, however, tubular delivery concepts should not be rejected; they can also offer advantages in stent-like applications [23,38,39].

### 3.4. Modified USP 4 Dissolution Testing

**Figure 6:**
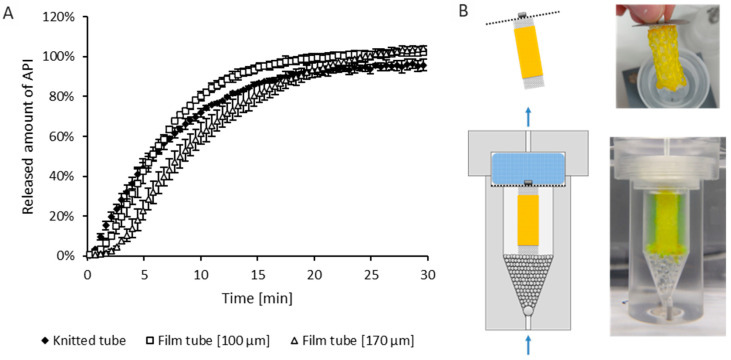
(**A**): Results of the release test of the knitted tube against film tubes of different thicknesses in the flow cell. (**B**): Schematic illustration of the modified flow cell with a special holder made of open-pore sponge and photo of the arrangement at insertion of the knitted tube and after one minute of testing.

The melt-extruded, spun and knitted mesh-like samples used for the dissolution tests had a surface of about 94 cm^2^, the film tubes had a surface of about 32 cm^2^. The time t = 0 was defined as the time when the first drop of medium flowed back into the reservoir. It can be seen from both the release curve and in Figure 6B that drug release occurred immediately at the start of the experiment. The photo of the flow cell taken at time t = 0 also clearly shows that the entire space of the flow cell is slightly yellowish in color, indicating that a non-negligible release has already taken place.

The open-pore sponge structure was able to provide some structuring assistance in the test setup but could not completely prevent very rapid release due to the large surface area of the dosage form and the fact that drug and polymer components also sank to the bottom of the release cell. Release was very rapid in all cases and was complete in less than 30 min.

The extremely large surface area of the fiber-based dosage form is also suitable for applications of poorly water-soluble active ingredients and could be further improved by producing much thinner fibers [40,41,42]. However, in combination with other excipients and thermoplastic polymers, the general advantages of melt extrusion in terms of improved solubility may come into play, in addition to the interesting possibilities of the filamentous dosage form. Soluplus was processed by electrospinning by Nagy et al. to compare it with melt extrusion. As a melt-extrusion-based fiber production technology was not yet established in pharmaceutical science, they produced thick extrudates and processed them into a fine powder [43]. 

The system we have developed allows for the first time in pharmaceutical science the melt-extrusion-based production of fibers, which will be tested against the electrospinning process in future research projects. The potentially large surface area of melt-extruded fiber-based ASDs and the ability to combine different functional and drug-loaded fibers during processing offer both biopharmaceutical and stability advantages [41,44]. However, it would also be possible to significantly prolong release from fibrous dosage forms, for example, by using polymethyl methacrylate ethyl cellulosse or other polymers used in applications such as medical sutures or implants. Consistent further development could result in a simple modular system of different functional fibers that can be used depending on the challenge of the active ingredient or application site and thus accelerate formulation development [45].

Excipients that were previously inaccessible to the electrospinning process could be made accessible by combining the melt extrusion process with appropriate further processing to create tailor-made drug release concepts. The advantages of smart textiles in particular offer extraordinary potential. For example, they can be triggered by electrical voltages, release targeted drugs, undergo mechanical changes or alter their structural behavior [28].

## 4. Conclusions

Existing approaches to produce hot-melt extruded pharmaceutical fibers and yarns were further developed and initial difficulties were overcome, so that the potential for the scalability of hot-melt extruded fibers and yarns loaded with active ingredients is given and further steps for consistent automated production at critical points could be defined.

In addition to investigations into quality control, tests were carried out on the further processing of the active ingredient-loaded fibers, with the initial focus on knitting the fibers into simple tubular structures. In the future, functional textile processes will also be used for the development of innovative dosage forms to realize further advantages of functionalized textile dosage forms, e.g., using shape-memory-based approaches, in addition to the advantages of using fiber-based dosage forms, e.g., to address the variability of the transit behavior of oral dosage forms.

To investigate the release behavior, a flow cell was modified so that mesh-like material could be tested. Particularly fast-dissolving single fibers, which are occasionally described and used in the literature to improve the solubility rate, also showed a very fast disintegration time in the experiments carried out here. As a result, disintegrated and swollen components of the fiber-based model dosage form were deposited on the ground of the flow through cell during release only a few seconds after the start.

The production of fiber-based dosage forms, especially in conjunction with melt extrusion, offers great potential for the development of innovative dosage form concepts. Innovative ways of processing fibers have already been described in the textile industry; they could be established in the pharmaceutical industry and, in conjunction with the possibilities of melt extrusion already established in the pharmaceutical industry, could open very interesting options for the development of dosage forms in the future, taking biopharmaceutical challenges into account.

## Data Availability

The data that support the findings of this study may be available on request from the corresponding author C.R., depending on requested information.

## Supplementary Materials

The following supporting information can be downloaded at: https://www.mdpi.com/article/10.3390/pharmaceutics16081103/s1, Table S1: Machine parameters to produce active ingredient loaded fibers.
