# Supplementary material for: Scalability of API-Loaded Multifilament Yarn Production by Hot-Melt Extrusion and Evaluation of Fiber-Based Dosage Forms
Source: Pharmaceutics. 2024 Aug 22;16(8):1103. doi: 10.3390/pharmaceutics16081103 (PMC11360357; doi:10.3390/pharmaceutics16081103)
Supplement: Supplementary file 1 [file pharmaceutics-16-01103-s001.zip › pharmaceutics-3141352-supplementary.pdf]

Table S1. Machine parameters to produce active ingredient loaded fibers.

<table>
<tr><th></th><th>Flat bottom dosing unit[1]</th><th>Hot melt extrusion[2]</th><th>Take of belt[3]</th><th>Spinning unit</th><th>Take-up roll[4]</th><th>Break - Material spool / Buffer tube[5]</th></tr>
<tr><td>Pre feeding</td><td>1%</td><td rowspan="4">Inlet: 20 °C
Z1: 80 °C
Z2: 150 °C
Z3: 165 °C
Z4: 165 °C
Z5: 165 °C
rpm: 150</td><td>/</td><td>/</td><td>/</td><td>/</td></tr>
<tr><td>Ramp up</td><td>1%</td><td>1.5 m/min</td><td>/</td><td>56.5 m/min</td><td>/</td></tr>
<tr><td>Yarn production (Standard)</td><td>1%</td><td>7.5 m/min</td><td>560 rpm</td><td>/</td><td>0.5 N</td></tr>
<tr><td>Spool changing</td><td>1%</td><td>7.5 m/min</td><td>560 rpm</td><td>/</td><td>0.5 N</td></tr>
<tr><td>Yarn production (Maximum)</td><td>2%</td><td>Inlet: 20 °C
Z1: 80 °C
Z2: 150 °C
Z3: 165 °C
Z4: 165 °C
Z5: 165 °C
rpm: 200</td><td>15.0 m/min</td><td>800 rpm</td><td>/</td><td>0.5 N</td></tr>
</table>

1. The use of a desiccant additive at both the flat-bottom feeder and the transition to the melt extruder, which always kept the humidity below 20% RH.
2. Zone 5 consists of a 90° stainless steel deflector with a thin multifilament die plate in front.
3. Modified with a stainless-steel pressure roller to stretch the material.
4. The speed is the maximum possible, the motor is connected to an elongation slip, so that the yarn material to be conveyed defines the maximum possible speed of the take-up roller.
5. The speed is determined passively from the speed of the spinning unit, delayed by a brake to ensure a constant material flow.
